# CORTO: The Celestial Object Rendering TOol at DART Lab

**DOI:** 10.3390/s23239595

**Published:** 2023-12-03

**Authors:** Mattia Pugliatti, Carmine Buonagura, Francesco Topputo

**Affiliations:** Department of Aerospace Science and Technology, Politecnico di Milano, 20156 Milan, Italy; carmine.buonagura@polimi.it (C.B.); francesco.topputo@polimi.it (F.T.)

**Keywords:** data generation, minor bodies, tool, image processing, image validation, space exploration

## Abstract

The Celestial Object Rendering TOol (CORTO) offers a powerful solution for generating synthetic images of celestial bodies, catering to the needs of space mission design, algorithm development, and validation. Through rendering, noise modeling, hardware-in-the-loop testing, and post-processing functionalities, CORTO creates realistic scenarios. It offers a versatile and comprehensive solution for generating synthetic images of celestial bodies, aiding the development and validation of image processing and navigation algorithms for space missions. This work illustrates its functionalities in detail for the first time. The importance of a robust validation pipeline to test the tool’s accuracy against real mission images using metrics like normalized cross-correlation and structural similarity is also illustrated. CORTO is a valuable asset for advancing space exploration and navigation algorithm development and has already proven effective in various projects, including CubeSat design, lunar missions, and deep learning applications. While the tool currently covers a range of celestial body simulations, mainly focused on minor bodies and the Moon, future enhancements could broaden its capabilities to encompass additional planetary phenomena and environments.

## 1. Introduction

CORTO is a tool that has been under development since the summer of 2020 by the Deep-Space Astrodynamic Research and Technology (DART) group (https://dart.polimi.it/, accessed on 8 August 2023) at Politecnico di Milano. The tool’s objective is to enable the high-fidelity, flexible, and simple generation of artificial image–label pairs of celestial bodies that can be used both to design, validate, and test Image Processing (IP) and visual-based navigation algorithms.

The motivations behind the tool development are multiple and address pressing issues faced by the authors of this work in research and industrial contexts.

First, open datasets of real imagery of close-up views of celestial bodies such as planets, moons, asteroids, and comets are scarce. This is due to a combination of different limiting factors: (1) We currently do not have an extensive sample of visited bodies in the Solar System, especially concerning asteroids and comets. (2) The existing datasets are limited by the mission geometry (e.g., in the case of flybys, often only a single face of the body is imaged at high resolution), posing stringent viewing and illumination conditions on the existing images. (3) Not all datasets from previous missions are publicly available (different space agencies have different dissemination strategies). Even when they are released for use by the broader engineering community, they are often not released shortly after arrival. This delay is due to embargo reasons and priority given to scientific investigations, introducing a delay into the availability of the images by researchers interested in visual-based applications.

In the history of space exploration, we have imaged at high resolution and investigated all major celestial bodies, but only a scarce amount of minor bodies such as asteroids and comets. Only 12 missions have been designed as flyby missions for asteroids, while an additional 10 missions have been designed as flyby missions for comets. This corresponds to a preliminary characterization of 24 different minor bodies (17 asteroids and 7 comets), some of which have been imaged during incidental flybys. The latter generates more data but requires a complex design, limiting the number of these types of missions. Only 6 missions have ever successfully orbited around a minor body, for a total of 7 bodies investigated up close. These are in chronological order of year of arrival: 433 Eros in 2002 with NEAR Shoemaker [1], 25143 Itokawa in 2005 with Hayabusa-I [2], 67P/Churyumov-Gerasimenko in 2014 with Rosetta [3], 4 Vesta in 2011 and Ceres in 2015 with Dawn [4], 162173 Ryugu in 2018 with Hayabusa-II [5], and most recently 101955 Bennu in 2018 with Osiris-Rex [6].

Second, to design, validate, and test image processing algorithms that use celestial bodies as targets, a common approach is to generate and use comprehensive high-fidelity datasets. Due to the lack of control over the viewing and illumination conditions of real-mission images, creating a digital model of the target body is often preferred to explore different environmental setups. An established approach is thus to artificially simulate the visual environment with the fidelity required by the algorithm to complement the existing data. This can be performed in two ways. When synthetic datasets are made out for existing and well-known bodies, they can augment existing images with additional geometric and illumination conditions. On the other hand, considering bodies that have never been imaged, synthetic datasets can be used to realistically represent them, providing a powerful tool for mission designers.

Third, as an increasing number of image-processing algorithms are being explored with data-driven design, a critical drawback exists for their adoption for space applications. These algorithms are often data-hungry, requiring labeled data for tuning and training, so their development clashes with the lack of data that characterizes space applications, particularly minor bodies. Moreover, supervised methods do not simply need data, but also associated labels representing quantities of interest that could be inferred from images. Concerning this work, the authors consider a label a quantity that is directly or indirectly represented in the image that could be inferred by an IP algorithm. These could be related to spatial information (such as the camera poses, range, phase angle, and rotational state of the target body) or to intrinsic properties of the target body (such as depth, slopes, or morphological classes) that become encoded in the images. Unfortunately, these quantities are not often precisely available. For example, reconstructed positioning data from a real mission can be used to represent the flew trajectory with an estimation error and used as a label for navigation algorithms. However, applications such as semantic segmentation may require data (e.g., a pixel-per-pixel classification of the classes of morphological features of an image) that are far more complex to generate.

Fourth, at the time of development, tools capable of artificially recreating the environment of celestial bodies were neither open-source nor easily accessible by individual researchers. Given the capabilities these tools unlock, their access is often regulated via licensing or is generally kept confidential since they provide a strategic advance. Moreover, their development requires a substantial investment of time and competencies that may not match that of an image-processing designer. Consequently, there has been a proliferation of different useful tools, which has created the effect of isolating communities and avoiding sharing datasets and tools for image generation. Among the most notable ones:

- PANGU [7,8,9] stands for Planetary Planet and Asteroid Natural scene Generation Utility and is considered the state of the art in rendering celestial bodies. It is a tool with robust, long-lasting, and documented development designed by the University of Dundee for the ESA. PANGU supports various advanced functionalities and is extensively used as the industry standard for ESA projects involving visual-based navigation algorithms. However, access to the software is regulated via licenses and often requires direct involvement with an ESA project as a pre-requisite.
- SurRender (v.6.0) [10,11] is proprietary software by Airbus Defense and Space (https://www.airbus.com/en/products-services/space/customer-services/surrendersoftware, accessed on 8 August 2023) that has been successfully used in designing and validating various vision-based applications for space missions in which the company is involved. The software can handle objects such as planets, asteroids, stars, satellites, and spacecraft. It provides detailed models of sensors (cameras, LiDAR) with validated radiometric and geometric models (global or rolling shutter, pupil size, gains, variable point spread functions, noises, etc.). The renderings are based on real-time image generation in OpenGL or raytracing for real-time testing of onboard software. Surface properties are tailored with user-specified reflectance models (BRDF), textures, and normal maps. The addition of procedural details such as fractal albedos, multi-scale elevation structures, 3D models, and distributions of craters and boulders are also supported.
- SISPO [12] stands for Space Imaging Simulator for Proximity Operations and is an open-access image generation tool developed by a group of researchers from the universities of Tartu and Aalto, specifically designed to support a proposed multi-asteroid tour mission [13] and the ESA’s Comet Interceptor mission [14]. SISPO can obtain photo-realistic images of minor bodies and planetary surfaces using Blender (https://www.blender.org/, accessed on 8 August 2023) Cycles and OpenGL (https://www.opengl.org/, accessed on 8 August 2023) as rendering engines. Additionally, advanced scattering functions written in Open Shading Language (OSL) are made available (https://bitbucket.org/mariofpalos/asteroid-image-generator/wiki/Home, accessed on 26 of October 2023) that can be used in the shading tab in Blender to model surface reflectance, greatly enhancing the output quality.
- Vizard (https://hanspeterschaub.info/basilisk/Vizard/Vizard.html#, accessed on 15 November 2023) is a Unity-based visualization tool capable of displaying the simulation output of the Basilisk (v.2.2.0) [15] software (https://hanspeterschaub.info/basilisk/, accessed on 15 of November 2023). Its main purpose is to visualize the state of the spacecraft; however, it has also been used for optical navigation assessment around Mars [16,17,18] and can simulate both terrestrial and small body scenarios [19].
- The simulation tools illustrated in [20,21] implement high-fidelity regolith-specific reflectance models using Blender and Unreal Engine 5 (https://www.unrealengine.com/en-US, accessed on 8 August 2023). The tools can render high-fidelity imagery for close proximity applications, particularly about small bodies, focusing on the high-fidelity simulation of boulder fields over their surfaces.
- AstroSym [22], developed in Python, provides a source of images for closed-loop simulation for Guidance Navigation and Control (GNC) systems for landing and close-proximity operations around asteroids.
- SPyRender [23], also developed in Python, is used to generate high-fidelity images of the comet 67P for training data-driven IP methods for navigation applications.

These challenges motivated the need of the DART group to develop a simple-to-use, easy-to-learn tool that would enable researchers and students to generate realistic images either to train or to test their image processing algorithms. This effort resulted in the development of CORTO, which has primarily targeted applications around small bodies and the Moon.

The tool is designed with a modular structure using various software and libraries. The realism of the final images is intended to bridge the domain gap between synthetic and real images, providing a powerful tool to mission designers. It is also remarked that CORTO’s capabilities are not limited to the generation of high-fidelity images, but also to the generation of the corresponding labels accompanying such images. These labels are fundamental in validating and testing IP algorithms. They encompass the creation of segmentation masks for the entire celestial body, both with and without accounting for shadows, the identification of boulders, terminator lines, crater rims, and the generation of depth and slope maps. Such a comprehensive set of labels associated with an image represents a significant innovation compared to previously described image generation tools, which often limit themselves to only a few specific labels. These labels are critical for designing and testing data-driven models, emerging as promising space application approaches. Moreover, CORTO introduces an innovative method for validating synthetic images with real ones, going beyond the conventional evaluation of intensity histograms. In this work, the main functionalities of CORTO are described in detail for the first time. Great care is also put into showing the validation of the tool’s output and the applications in which CORTO is currently being used. The tool is openly available at https://github.com/MattiaPugliatti/corto (accessed on 15 of November 2023) while it is being maintained and further developed.

The rest of the paper is organized as follows. In Section 2, the architecture and functionalities of CORTO are described in detail. In Section 3, the validation procedure adopted to assess the tool’s capabilities is illustrated. In Section 4, the missions and projects in which the tool has been deployed are listed as application cases. Finally, in Section 5, some considerations for future developments are listed.

## 2. Architecture of the Tool

CORTO is at the core of the tools developed and used at the DART lab to simulate the visual environment around celestial bodies. CORTO can be used to generate image–label pairs both online, with GNC systems connected in closed-loop, or offline to create datasets for statistical analysis or design. The tool’s core is the Blender software (v.4.0), an open-access rendering software that is easy to learn, with a large community, and supports Python scripting. The main functionalities are handled in Python, while Matlab, Simulink, and SPICE are used within the tool.

**Figure 1:**
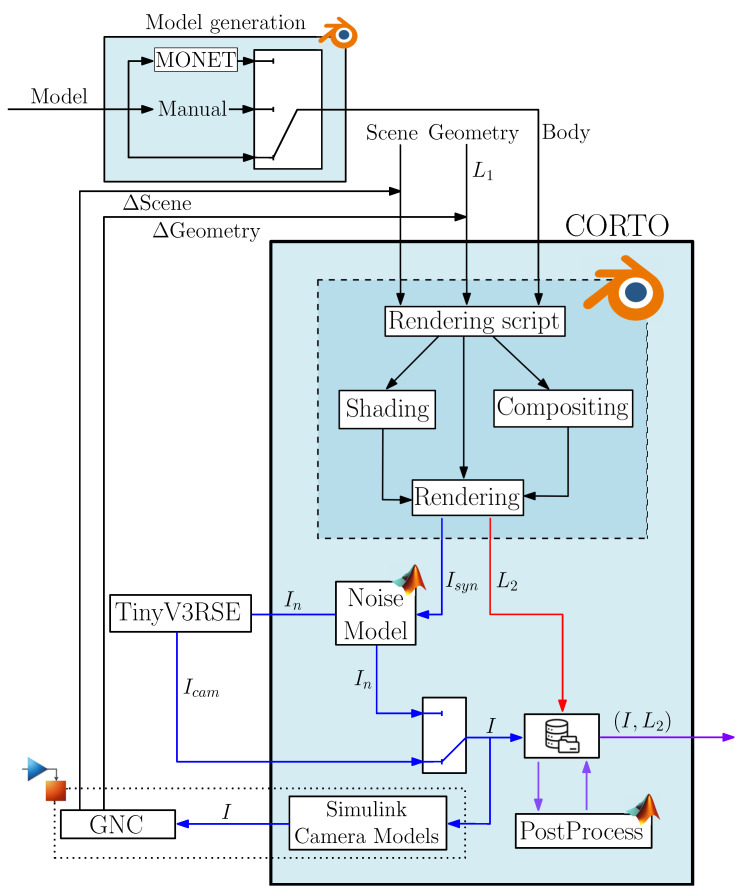
High-level architecture of CORTO and other tools used at the DART lab.

The general architecture of CORTO, as well as its relationship with other tools and simulators of the DART group, is illustrated in Figure 1. The inputs to CORTO are *Scene*, *Geometry*, and *Body* configuration files, while the outputs of the tool are images (*I*) and labels (*L*). The tool can be used in open-loop by using a set of pre-defined configuration files containing all the setup to be used for renderings or in closed-loop by updating only the *Scene* and *Geometry* inputs after the generation and processing of each image. Currently, the tool is not designed for real-time rendering but focuses on dataset generation and delayed closed-loop simulations.

A critical functionality of CORTO is the capability to generate labels associated with the images. These are divided into two groups: $L_{1}$ and $L_{2}$. $L_{1}$ represents quantities also used as input in Blender to position the objects in the scene (e.g., the poses of all the bodies and derivatives quantities such as the range, phase angle, and others). $L_{2}$ represents labels generated in Blender during the rendering process (e.g., pixel classes, depth maps, slope maps, and others). Both sets of labels can be used and coupled with images, but it is important to distinguish between them, as they are of different nature.

Below, the flow of how to use the tool is now exemplified, while the core blocks are explained in detail in the following subsections. The starting point is the *Model generation* block in Figure 1, which takes as input a rough mesh model saved as an “.obj” and generates the *Body* input for CORTO. This block can be used to augment an existing object into a high-resolution one with morphological features of interest.

**Figure 2:**
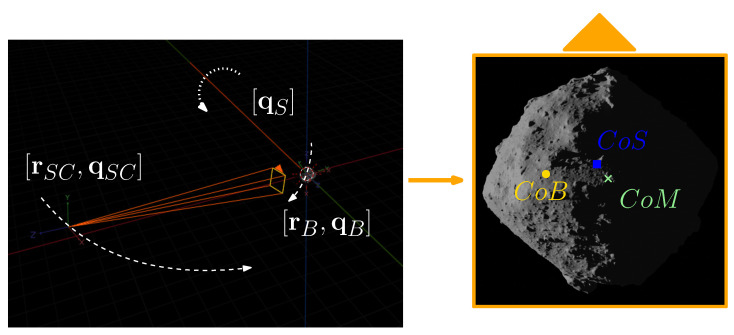
Example of scene generation from *Geometry* input. Position and orientation of the objects in the scene in Blender (**left**) and the image obtained (**right**).

To operate, CORTO needs two other inputs, referred to as *Geometry* and *Scene*. The *Geometry* input consists of a configuration file in which the poses of the objects involved in the scene are handled. These include the spacecraft position ($r_{SC}$) and orientation ($q_{SC}$), the body position ($r_{B}$) and orientation ($q_{B}$), and the Sun orientation ($q_{S}$), all in a shared reference system, as it is possible to see in Figure 2. Finally, the *Scene* configuration file contains data about camera, material, and illumination properties of the Sun’s lamp.

The *Body*, *Geometry*, and *Scene* inputs are then read by a Python rendering script. The script is the core component of CORTO and is used to set up all necessary configurations for the *shading*, *compositing*, and *rendering* tabs in Blender. The script manages the renderings and the generation of the image–label pairs, denoted as $I_{syn}$ and $L_{2}$. An example of scene rendering from CORTO input is illustrated in Figure 2. Note that the Sun’s geometrical settings are commanded only by its orientation $q_{S}$ and are invariant to its position. For simplicity, it can be conveniently fixed to the center of the target body.

After generation, images can pass into the *Noise model* block implemented in Matlab, which adds artificial noise to the synthetic images $I_{syn}$. The noise can represent both camera and environmental disturbances and its addition can be selectively disabled. This is particularly relevant when performing Hardware-In-the-Loop (HIL) simulations since the camera noise can be modeled directly by the stimulated camera. For this purpose, the Tiny Versatile 3D Reality Simulation Environment (TinyV3RSE) facility [24] can be used. This facility consists of a screen–collimator–camera setup in which a physical camera is stimulated by a high-resolution screen. The purpose of this facility is to include a camera within the loop of the IP algorithm and to generate noisy images with it.

The output image *I* after noise addition (either artificially generated or with an HIL setup) can then follow two routes depending on the open-loop or closed-loop settings. In an open-loop scenario, the image is saved in a database. In a closed-loop scenario, the images are transferred to a module in Simulink that acts as a virtual camera sensor that stimulates a complete GNC subsystem. This approach uses transmission control/internet protocols to transmit images and flag commands that generate the subsequent spacecraft pose, repeating the entire cycle.

Finally, a postprocessing step can be applied to prepare the image–label pairs to constitute a dataset. This is especially relevant for data-driven algorithms (that may require data augmentation, cropping, and resizing) but also applies to traditional algorithms.

Domain randomization is a powerful technique that allows for a large variation in the possible image space. In this work, this is intended in terms of the appearance of the input image. Properly tuning the settings within the rendering, noise modeling, and postprocessing blocks allows to obtaining of geometrically identical samples of the same image with randomized appearance. This technique can be used as a data augmentation technique to enable a data-driven model to become robust about noise, albedo, illumination, material properties, and body position within the input image.

**Figure 3:**
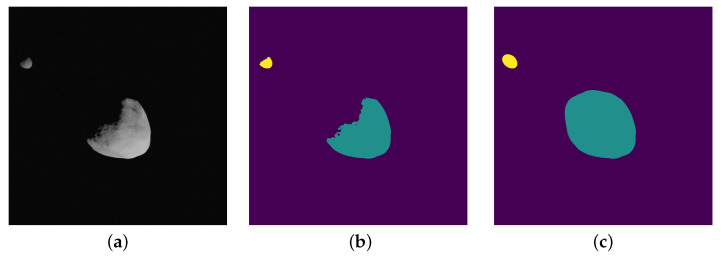
Image and associated masks for the background (purple), Didymos (green), and Dimorphos (Yellow) classes. (**a**) Input grayscale image. (**b**) Masks with shadows. (**c**) Masks without shadows.

As explained before, the core capability of CORTO is not only that of generating high-fidelity images but also of generating labels accompanying such images. What follows is a set of examples of labels that can be obtained from CORTO. In Figure 3, it is possible to see an image of the Didymos system and associated pixel labels that could be used for object recognition to distinguish between Didymos, the primary body, and Dimorphos, its secondary. In Blender, these masks can be obtained by properly setting the object’s pass indices. These are optional values that are tracked by the raytracing procedure during rendering.

**Figure 4:**
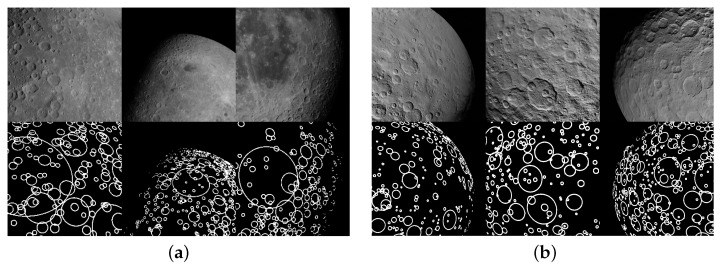
Example of images with crater labels obtained in Blender by elaborating a high-fidelity texture map from the Robbins and Zeilnhofer datasets. (**a**) Moon craters. (**b**) Ceres craters.

In Figure 4, it is possible to see different labels about craters on the Moon and on Ceres generated using high-fidelity texture maps obtained from the Robbins [25] crater dataset (https://astrogeology.usgs.gov/search/map/Moon/Research/Craters/lunar_crater_database_robbins_2018, accessed on 8 August 2023) and the Zeilnhofer [26] crater dataset (https://astrogeology.usgs.gov/search/map/Ceres/Dawn/Craters/ceres_dawn_fc2_craterdatabase_zeilnhofer_2020_v2, accessed on 8 August 2023).

**Figure 5:**
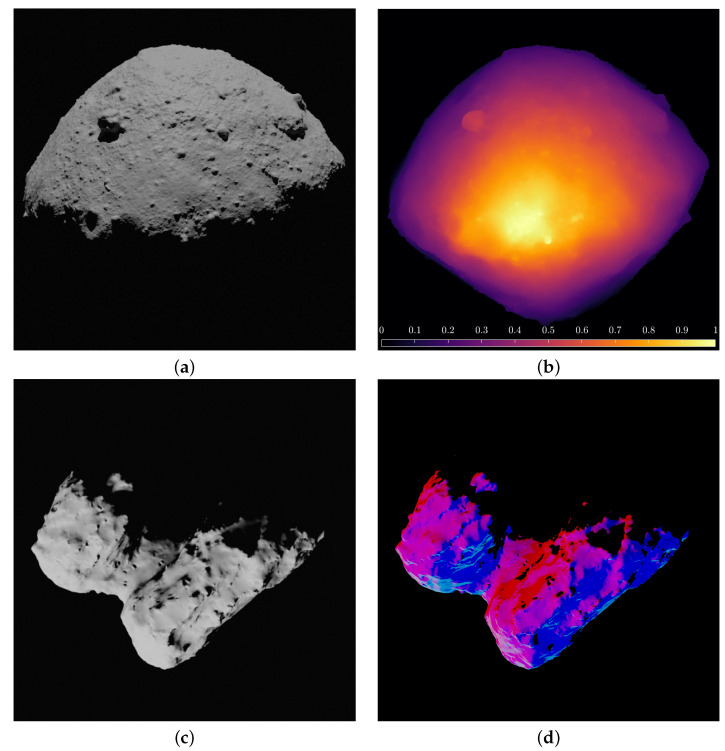
Examples of the depth map and slope labels with asteroid Ryugu and comet 67P. (**a**) Input image of Ryugu. (**b**) Depth map label of Ryugu. (**c**) Input image of 67P. (**d**) Slope label of 67P.

In Figure 5, examples of depth-map and slope labels are generated for asteroid Ryugu and comet 67P. Both are generated simply using the Cycles rendering engine in Blender and adding the *Depth* (or *Depth*) and *Normal* datatypes as outputs of the rendering engine.

**Figure 6:**
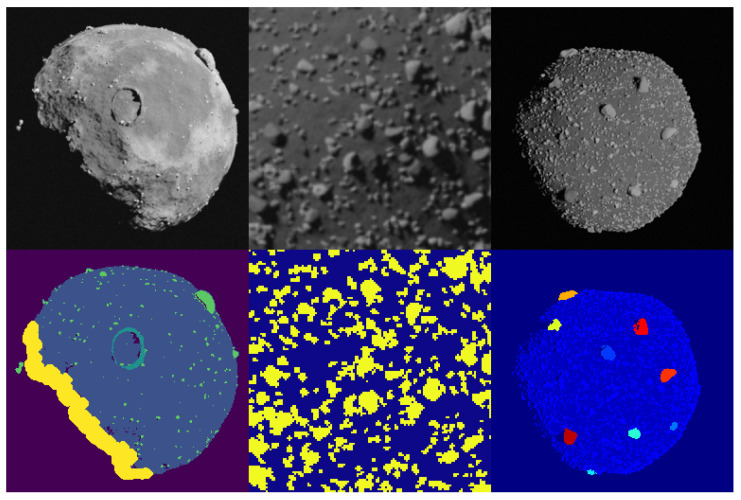
Examples of masks that can be generated for semantic segmentation applications.

Finally, in Figure 6, it is possible to see different examples of segmentation maps that can be used generated about small bodies that exploit different pass indices strategies to obtain multi-layers maps, single class maps (boulders-surface) and hierarchical ones (background, surface, small boulders, and prominent boulders divided by single identifiable color codes). The full procedure adopted to generate these masks is not explained in this work. However, the interested reader is directed to [27,28] for extensive explanations on how to generate the multi-layer and hierarchical segmentation masks.

### 2.1. Object Handling

One of the most important inputs to CORTO is the shape model of the target body. Depending on the observation technique used to generate it, available shape models can be rough (e.g., when observed with radio telescopes from Earth or during a flyby) or accurate (e.g., when observed during a rendezvous mission).

Each model passes through the *Model generation* block independently from the source, following one of three possible paths. If the model is already accurate for the task considered, it can be passed as it is directly as an “.obj” to CORTO. However, in many cases, the shape model is not sufficiently accurate for the task considered. In these cases, both manual and procedural modifications are dedicated to refining the model mesh and introducing morphological features such as roughness, craters, and boulders over the surface. These are added in Blender, but in principle, any software capable of modifying a 3D mesh can be used.

When manual refinement is performed, details are arbitrarily added over the surface. For example, the mesh can be thickened, surface roughness can be achieved using noise elements as textures, and craters and boulders can be introduced using Blender’s built-in tools. This allows objects to be placed in the desired positions and appearance. The entire process is manual and artistic, does not allow for reproducibility, and requires skills from the user in working with 3D objects [27].

Lastly, the model can be procedurally modified by using Minor bOdy geNErator Tool (MONET) [29] designed at DART. MONET takes the model as input, automatically refines and smoothes the mesh, and introduces morphological features from user-defined input values. By default, MONET is capable of realizing two different categories of minor bodies, namely rocky bodies (characterized by a large number of different-sized boulders on the surface) to simulate rubble-pile asteroids and comet-like bodies (whose surface exhibits the alternation of very smooth and rough regions typical of comets). Adjusting the tool’s settings makes it possible to model the properties of a variety of minor bodies in a complete procedural approach.

### 2.2. Rendering

The rendering procedure is handled by a Python script but executed in Blender. Two different engines are used to generate the renderings: Cycles and Eevee. The former uses path-tracing algorithms and is considered more photo-realistic but resource-intensive. The latter uses simplified light environments and can render simpler scenes accurately in a shorter time. Considering the photorealism needed from the images, the type of labels desired, and the rendering speed with the available hardware, the user might prefer one of the two rendering engines.

**Figure 7:**
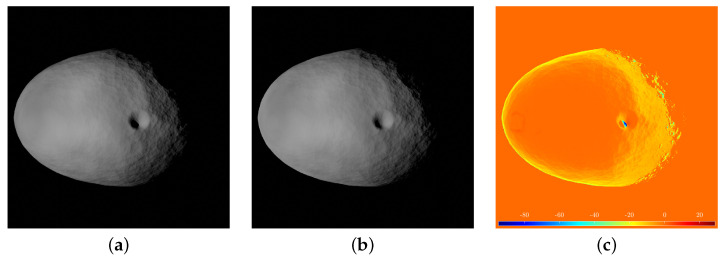
Example of different rendering engines used in Blender. (**a**) Cycles rendering. (**b**) Eevee rendering. (**c**) Pixel intensity difference.

In Figure 7, it is possible to see the rendering difference in the same scene of the Dimorphos asteroid when using Cycles and Eevee.

When building the body properties via the input parameters across the different shading, compositing, and settings tabs in Blender, it is essential to define the requirements of the final image that best reflect the needs of the processing algorithm that will use such images. These will drive critical choices on the body properties and rendering settings to be used.

For example, the surface of a celestial body could be rendered using using the standard Principled Bi-directional Scatter Distribution Function (PBSDF) implemented in Blender, combining it with a texture map, or employing ad hoc scattering laws coded in OSL, as in [12]. Texture maps, however, are a scientific product of a mission and might not be available for the body of interest. Moreover, even if available, they could be generated accurately only for a portion of the body or with offset phase angles that may introduce spurious shadows into the images, invalidating their photorealism. At the same time, their high variability and close resemblance to the real surface albedo may be critical for a feature-tracking algorithm that may prefer them over a plain mesh surface modeled with a combination of scattering functions. Another alternative currently under investigation and based on recent works [30] focuses on assigning single scattering properties to each boulder scattered across the surface of a model of an artificial asteroid. At this stage of development, however, this option is computationally intensive and yet to be formalized in the nominal pipeline of CORTO. On the other hand, if the target application is a limb-based algorithm (or any other algorithm using global properties), the designer may neglect texture maps altogether and focus on the global appearance of the body and the simulation of the correct scattering function.

These considerations are crucial in designing an appropriate artificial environment for the task considered. The user might consider the pros and cons of each modeling strategy and select the one that best fits the algorithm-specific objectives and design. Currently, the surface of the object in CORTO can be simulated using the standard PBSDF, PBSDF in combination with texture maps, or a set of defined scattering functions [31] introduced using OSL. Note that these functions are the same developed in (https://bitbucket.org/mariofpalos/asteroid-image-generator/wiki/Home, accessed on 15 November 2023) and are: (1) Lommel–Seeliger [32], (2) ROLO [33], (3) Akimov [34,35], (4) Linear Akimov [31], (5) Lunar Lambert [36], and (6) Minnaert [37].

### 2.3. Noise Modelling

Adding noise into the synthetic images generated by CORTO is an important step to make them more similar to real images acquired by navigation sensors onboard previously flown missions.

**Figure 8:**
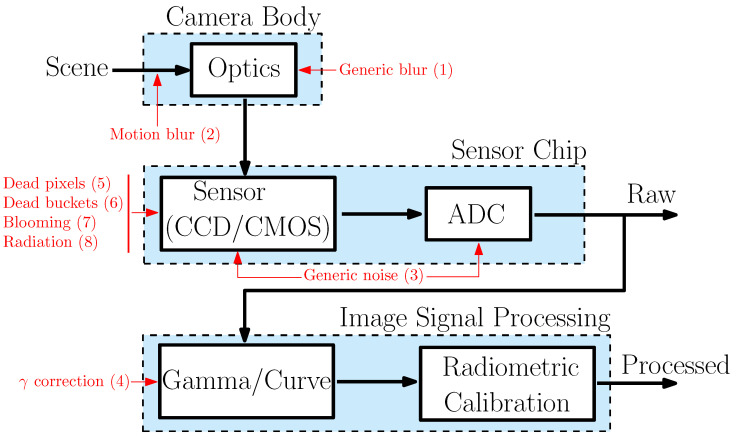
Noise modeling block in CORTO. Adapted from [38]. Different sources of noise are represented in red with the order they are applied in the script.

The noise block in CORTO does so with the methodology summarized in Figure 8, which has been adapted specifically for visual-navigation space cameras from the noise modeling in [38,39]. For this reason, the block is currently applicable to grayscale images only, and shutter or aperture effects are not considered assuming typical space cameras.

The block is currently implemented in Matlab and the different sources of noise that the user can parametrize are represented in red in Figure 8, spread into eight different steps. In (1), generic blur is introduced with the use of a Gaussian filter from the *imgaussfilt* function, while motion blur is simulated in (2), generating a specific motion filter with the *fspecial* function. In (3), generic noise is added with the *imnoise* function. In (4), gamma correction is performed over the image using the *imadjust* function. After these phenomena are modeled, sensor effects are introduced in (5), (6), and (7) by altering the pixel values in the images, removing single pixels, entire rows and columns, or saturating pixel content. Finally, in (8), radiation effects are introduced simulating randomized lines spanning over the image saturating pixel’s content.

**Figure 9:**
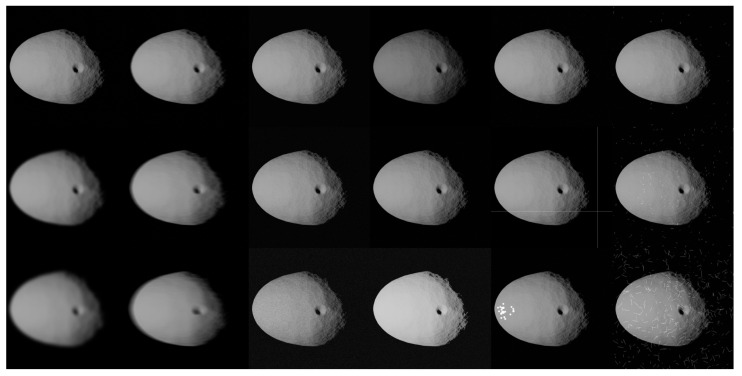
Example of different sources of noise, low to high from top to bottom. From left to right: Generic blur, motion blur, generic Gaussian noise, gamma correction, sensor effects (from top to bottom: dead pixels, dead buckets, and blooming), and radiation effects.

The different sources of noise illustrated in Figure 8 are exemplified on an image of Dimorphos at low (top), medium (center), and high (bottom) values in the mosaic image in Figure 9 (except for dead pixels, dead buckets, and blooming effects, which are illustrated from top to bottom in the second column from the right).

**Figure 10:**
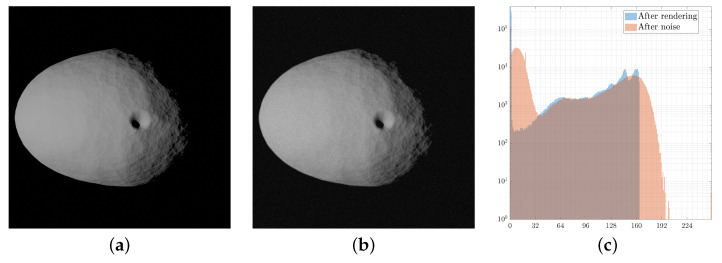
Effects of the application of noise to the synthetic images. (**a**) Synthetic image after rendering without noise. (**b**) Synthetic image after application of noise. (**c**) Histogram comparison with and without noise.

The effects of noise modeling are visualized in histogram form in Figure 10. These effects are important to change the image content of a clean synthetic sample toward a more realistic one.

Finally, it is mentioned that the noise modeling block can be used in two pragmatic approaches. First, one could mimic the target noise effects expected from a specific camera for a pre-designed mission. Second, one could also apply noise with statistical sampling from a pre-defined distribution to generate datasets with images with generic and realistic noise that is not typical of any sensor. The latter methodology tends to perform better with a data-driven approach in terms of domain randomization, increasing their robustness and applicability to real images.

### 2.4. Hardware-in-the-Loop

CORTO can also be used to provide synthetic images to the TinyV3RSE, an optical testbench facility. TinyV3RSE [24] is constituted by a high-resolution screen, a plano-convex collimator lens, and a camera.

The image generated by CORTO can be projected on the screen, stimulating the camera via the collimator, which is used to make sure the screen is imaged as seen from infinity. Calibration procedures ensure all the facility’s elements are centered and aligned [24].

The facility’s purpose is twofold. First, including an engineering model of the target camera can be useful in identifying unforeseen bottlenecks and preparing the correct interface. Second, the camera allows the transformation of an otherwise clean Blender image into a noisy one, with characteristics that would be similar to a real deployment.

**Figure 11:**
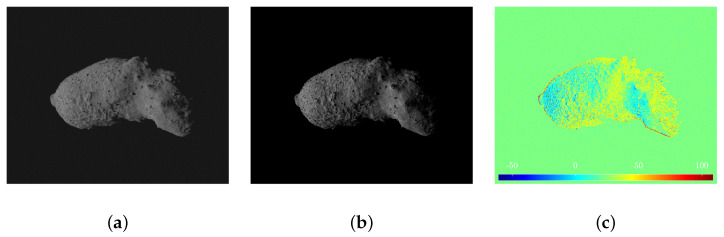
Geometric equivalence between synthetic and facility images. (**a**) Synthetic image. (**b**) Facility image. (**c**) Pixel intensity difference.

A comparison between a synthetic image and its equivalent image captured within the TinyV3RSE facility is illustrated in Figure 11. The two images are photometrically different (the camera–screen interaction introduces noise and photometric calibration is not performed in TinyV3RSE) but geometrically equivalent, as it is possible to see from the image difference in Figure 11.

### 2.5. Post-Processing

A postprocessing block in CORTO adapts the image–label pairs for the specific target application. This is particularly relevant when developing data-driven image processing methods, such as convolutional neural networks, that traditionally require constant size tensors for training, validation, and testing. Several operations may be performed in this block to transform an arbitrary resolution, noisy sample into a proper dataset sample.

**Figure 12:**
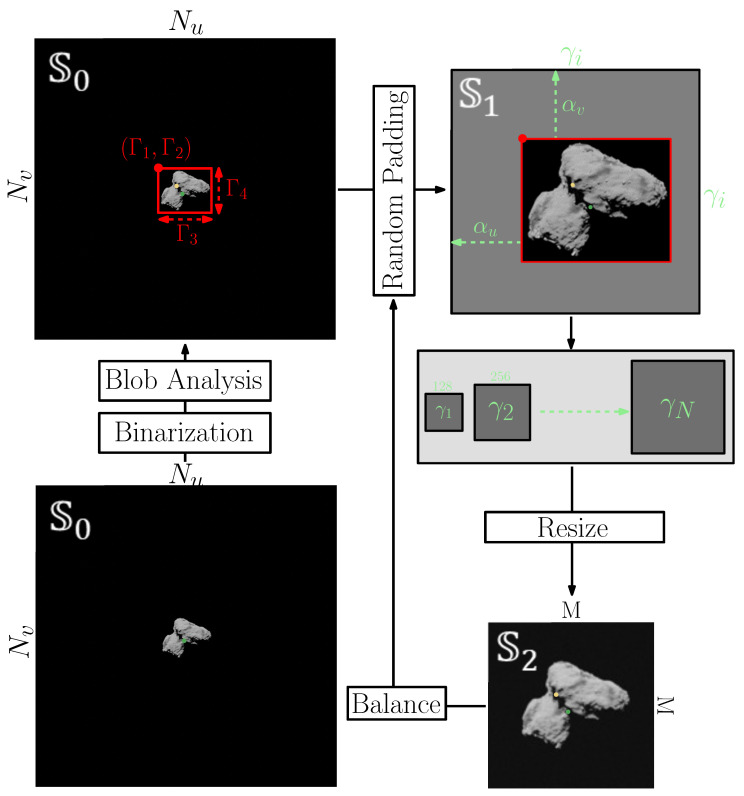
High-level architecture of the postprocessing pipeline.

The pipeline for the image–label postprocessing is illustrated in Figure 12. Currently, only labeling changes for the center of mass, center of brightness, and range of the body are supported, but other labels can be easily included in the pipeline if needed. Also, the pipeline processes squared images, but its extension to rectangular ones is trivial.

The original image obtained after rendering and noise addition is said to be defined in a $S_{0}$ space with a resolution $N_{u}\times N_{v}$. The image is binarized, and a simple blob analysis is performed. The N-th biggest blobs are identified and grouped together to form a single object with a bounding box Γ (defined by the top-left corner coordinates $\Gamma_{1}$ and $\Gamma_{2}$ and by the width $\Gamma_{3}$ and height $\Gamma_{4}$). Random padding of the image outside of the bounding box Γ is performed to transform the $\Gamma_{1}\times \Gamma_{2}$ bounding box into a square of resolution $\gamma_{i}\times \gamma_{i}$ in $S_{1}$ space. The padding is performed by randomly sampling two scalars $\alpha_{u}$ and $\alpha_{v}$ from uniform distributions, each ranging from 0 to the maximum value that would transform the rectangular $\Gamma_{1}\times \Gamma_{2}$ box into a square one of side $\gamma_{i}$. During padding, no new pixel content is generated, and the same pixel content from the original image in $S_{0}$ is instead retrieved. Also, note that the target size $\gamma_{i}$ of the image in $S_{0}$ can be selected by the user as a multiple of the final target size *M*. Finally, the image is resized and transformed into a M×M matrix, defined in $S_{2}$. Lastly, a final step can be performed to repeat the random padding process multiple times. This may be necessary if the user is interested in balancing the image appearance or its label distributions in the dataset and would like to implement different input distributions.

All these steps are part of a data augmentation strategy specifically designed for celestial bodies and their labels of interest. Traditional data augmentation strategies turned out to be limiting the capabilities to transform the image–labels pairs in a useful way. It is also mentioned that only the procedure for changing the image is illustrated in Figure 12, but the labels are changed simultaneously to ensure they can be correctly recovered. The steps to change the labels are illustrated in the Appendix A of this work.

At inference, it is possible to transform the estimated quantities from $S_{2}$ to $S_{0}$. The pipeline in Figure 12 can thus be easily implemented onboard: processing an image and transforming it from $S_{0}$ to $S_{2}$, performing inference and generating estimated labels in $S_{2}$, then finally transforming the labels as $S_{0}$ quantities.

This is possible because the pipeline has been implemented specifically for onboard implementation: All of the steps illustrated in Figure 12 can be performed onboard over each image acquisition to transform it into a processable input for an IP algorithm. This is possible by storing the various parameters used during processing illustrated in Figure 12. Moreover, the designed pipeline was necessary to remove large portions of the image carrying little information (the background), a specific property of images of celestial bodies, especially when seen from relatively far away.

Finally, thanks to the postprocessing strategy just described, generating multiple versions from the same input image with different image–label pairs is possible. This can be useful for performing data augmentation for generalization purposes and implementing domain randomization designs.

### 2.6. Reproduce Previously Flown Missions

Recreating images obtained from previously flown missions holds tremendous potential since it enables direct comparison with previously flown algorithms using the same type of input images.

For this reason, a tool has been designed to recreate images taken from previously flown missions. The tool uses the available metadata associated with the images to extract the epoch and, combining this with the camera properties and the spacecraft–body–Sun relative poses, generates a set of inputs that can be used in CORTO. The objects poses are retrieved from kernels using SPICE (https://naif.jpl.nasa.gov/naif/data.html, accessed on 8 August 2023). At the current stage of development, this tool can replicate images taken from missions led by major space agencies, such as the ESA, NASA, and JAXA.

Note that images reproduced with this tool are accompanied by the same attitude estimation and positioning errors reflected in the ephemerides. This, in turn, translates into small errors in the labels, which need to be considered.

## 3. Validation

Validating synthetic images of celestial objects with real ones is crucial in ensuring synthetic images’ accuracy, reliability, and applicability for various scientific and operational purposes.

While image histogram comparison is often used as the primary method to assess the similarity between synthetic and real images [40,41,42], it represents the satisfaction of a necessary but not sufficient condition. An image histogram represents the distribution of the image content across different intensities, but in doing so, it inevitably brings a loss of spatial information.

**Figure 13:**
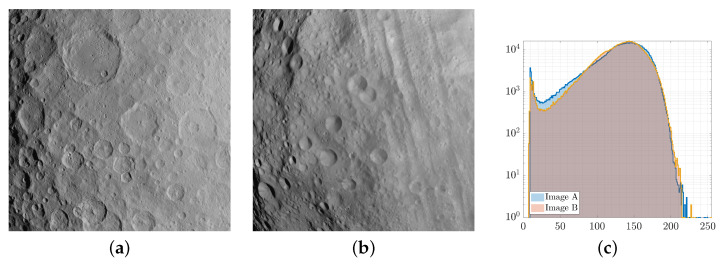
Image of Ceres (**a**) and Vesta (**b**) along with their overlapping histograms (**c**).

For example, in Figure 13c, two images of two different asteroids, namely Ceres and Vesta, are illustrated with their image histograms overlapped. These images have been captured by the Dawn [4] mission using the same camera. Although their histograms are similar and exhibit a consistent overlap, it cannot be concluded that the two images correctly represent the same scenery, showcasing how risky it is to adopt this criterion alone when assessing image similarity for validation purposes. The histogram overlap just describes a similarity in the pixel content of the image, which does not reflect a similar spatial distribution of such content.

Although it is important to check that similar images exhibit similar histograms, a systematic approach should be used instead that can better quantify image differences.

**Figure 14:**
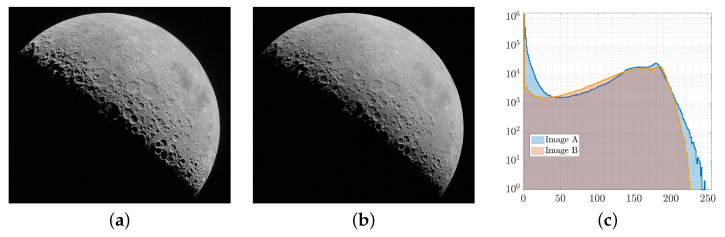
Real (**a**), synthetic (**b**) manually reproduced image of the Moon along with their overlapping histograms (**c**).

A manual approach would adjust the rendering settings and the relative body–camera–Sun poses. For instance, Figure 14 shows a manually reproduced image of the Moon seen with a full limb compared to one captured on the Orion spacecraft with a navigation camera (https://www.nasa.gov/image-feature/orion-gazes-at-moon-before-return-to-earth, accessed on 8 August 2023). Although this approach can yield faithful reproduction of real images, it demands a significant amount of time and introduces human errors. To overcome these limitations, a systematic approach is proposed instead in this work to validate the functionalities of CORTO.

### 3.1. The Validation Pipeline

This work proposes a validation pipeline as a systematic approach to evaluate image similarity considering both pixel intensity values and overall image structure.

**Figure 15:**
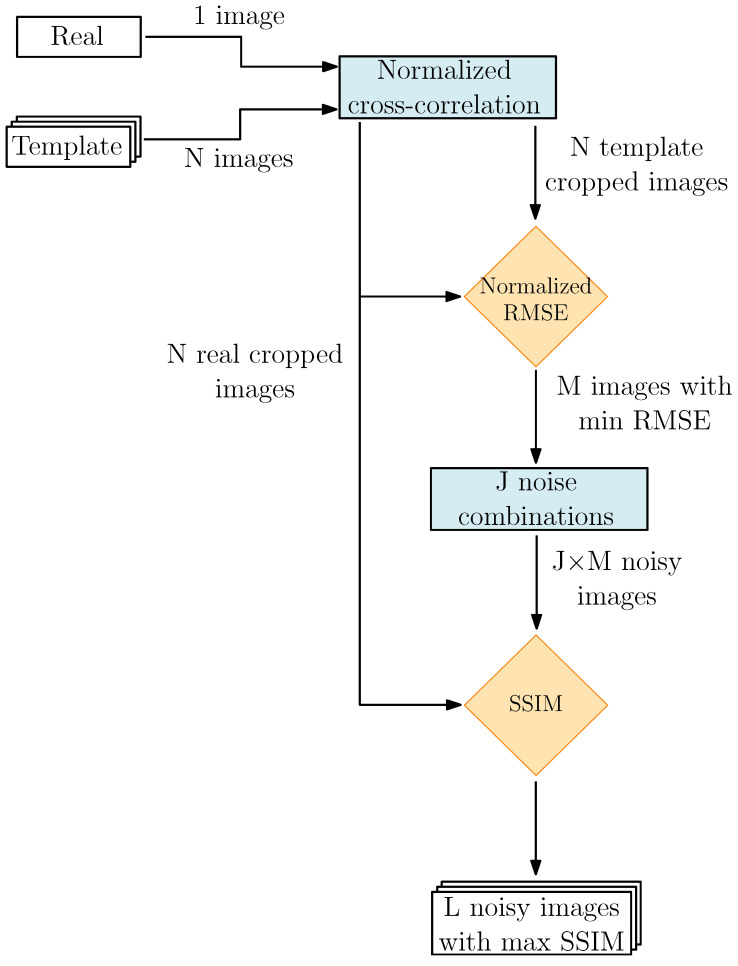
High-level architecture of the validation tool.

A schematic of the validation pipeline is represented in Figure 15. The pipeline inputs are the real image and *N* template images generated using varying settings in CORTO. These may include rendering, shading, material, surface, and light properties.

As depicted in Figure 15, the first operation involves a normalized cross-correlation [43] between the *N* templates and the real image to reduce the camera pose errors introduced by the state reconstruction, computed as:

(1) $$\gamma \left(u,v\right)=\frac{\sum_{x,y}\left[f\left(x,y\right)-{\bar{f}}_{u,v}\right]\left[t\left(x-u,y-v\right)-\bar{t}\right]}{{\left\{\sum_{x,y}{\left[f\left(x,y\right)-{\bar{f}}_{u,v}\right]}^{2}\sum_{x,y}{\left[t\left(x-u,y-v\right)-\bar{t}\right]}^{2}\right\}}^{0.5}}$$

where (x,y) denotes the pixel location, *f* is the real image, $\bar{t}$ is the mean of the template, (u,v) is the coordinates of the template center in the real image, and ${\bar{f}}_{u,v}$ is the mean of f(x,y) within the template region. Following this operation, the images are cropped to maximize the correlation, which is particularly significant for far-range observations, where the complete silhouette of the body is visible. The outcome of this process yields *N* cropped template images along with their corresponding cropped real images, such that each template has a corresponding real image with the same resolution. Subsequently, the cropped real and template images are compared with a Normalized Root Mean Square Error (NRMSE) [44], computed as:

(2) $$\mathrm{NRMSE}=\frac{\sqrt{\frac{1}{d}\sum_{x^{\prime },y^{\prime }}{\left[t\left(x^{\prime },y^{\prime }\right)-f\left(x^{\prime },y^{\prime }\right)\right]}^{2}}}{d}$$

where *d* represents the number of pixels and $\left(x^{\prime },y^{\prime }\right)$ denotes the pixel location in the cropped images. The normalization approach has been selected because the previous correlation step generates different-sized images. Consequently, the value of the RMSE is scaled to be independent of the image size for a better comparison.

The first *M* images (with the lowest NRMSE values) are selected because of the similarities in pixel intensities. Once the best ideal synthetic images are selected, accounting for noise inherent to the environment and camera errors is necessary. Because of this, *J* different noise combinations are applied to each of the *M* images. Specifically, the considered noise source includes Gaussian noise mean and variance, blur, and brightness. The assumed noise values are specified in Table 1, resulting in 192 combinations.

As a result, a total of J×M noisy images become available. Lastly, a second comparison step uses the Structural Similarity Index Measure (SSIM) [45]. This metric is employed because it considers the structural information embedded in the image, separating it from the influence of the illumination. The metric is defined as:

(3) $$\mathrm{SSIM}\left(x^{\prime },y^{\prime }\right)={\left[l\left(x^{\prime },y^{\prime }\right)\right]}^{\alpha }{\left[c\left(x^{\prime },y^{\prime }\right)\right]}^{\beta }{\left[s\left(x^{\prime },y^{\prime }\right)\right]}^{\gamma }$$

where *l*, *c*, and *s* represent the image’s luminance, contrast, and structural terms, respectively. The coefficients α, β, and γ are all set to 1 to ensure equal contribution. After evaluation by the SSIM, the *L* images with maximum SSIM are selected as best validation candidate.

### 3.2. Validation Examples

Using the validation pipeline described in the previous section, the tool’s capability is validated considering four minor bodies: Ceres, Vesta, Bennu, and 67P. These target bodies have been selected because their images are readily available from the Dawn [4], Osiris-Rex [6], and Rosetta [3] missions, and since they represent a diverse sample in terms of global shape and surface characteristics. Moreover, these bodies are representative of missions from three different space agencies (ESA, NASA, and JAXA), demonstrating the tool’s capability to work with different metadata formats.

Different strategies to represent the surface are investigated for each body: “OSL” if specific scattering laws (such as the one designed in [12] and presented in Section 2.2) are used, “PBSDF” if the standard Blender scattering function is used in the shader (without a texture), and “PBSDF + Texture” if an existing body texture has been used instead, coupled with the PBSDF.

Table 2 summarizes the total number of template images for each combination considered. Note that some cases are not considered in the pipeline (e.g., Vesta and 67P with Texture, or Ceres with only OSL or PBSDF) and these settings are not considered for representing these bodies, as the texture maps would not be available or the scattering functions alone would not be capable of yielding the desired fidelity. The template images from Table 2 are obtained by varying the illumination intensity of the Sun’s lamp while changing the albedo’s properties of the target body and the scattering function adopted. Finally, it is remarked that the case of asteroid Bennu has been investigated across all possible reflectance models since the existence of a high-quality and low phase-angle texture map of Bennu allows for such a detailed comparison.

**Figure 16:**
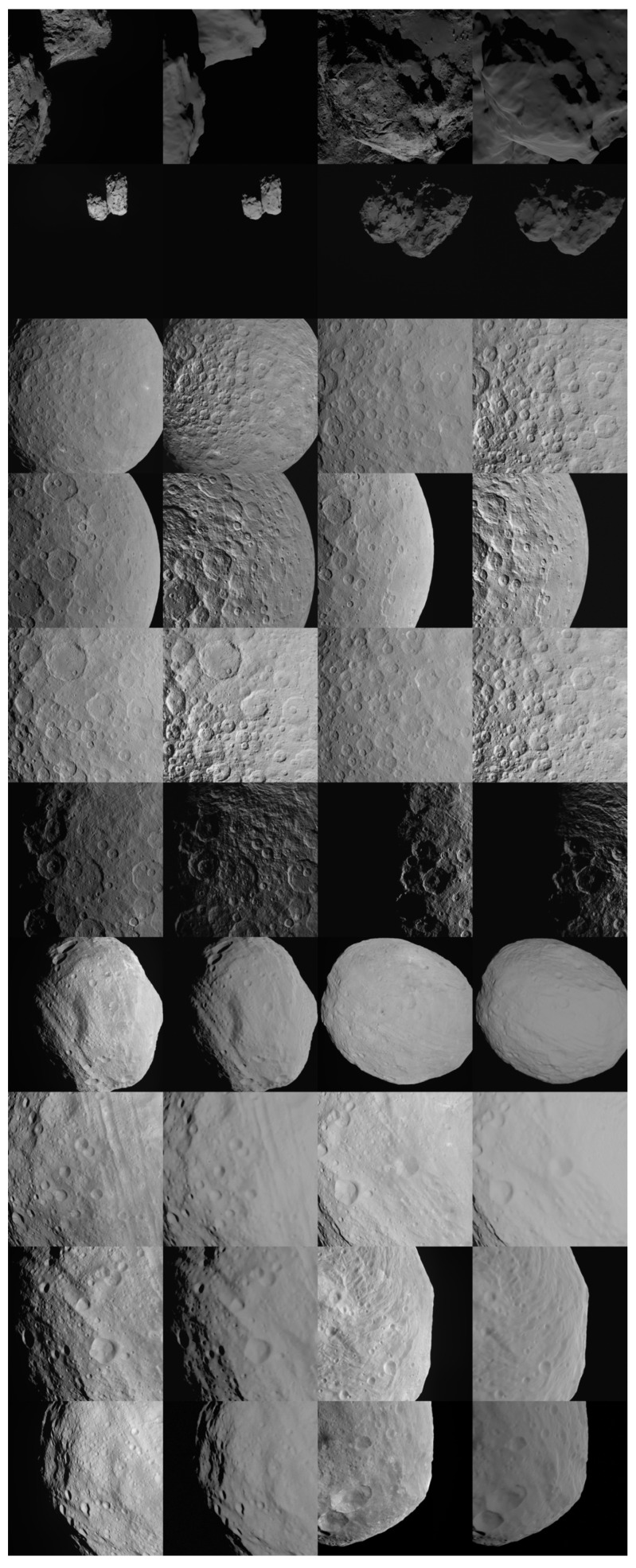
Mosaic view of synthetic and real images of 67P, Ceres, and Vesta. The first and third columns represent real images while the second and fourth ones images generated using CORTO.

**Figure 17:**
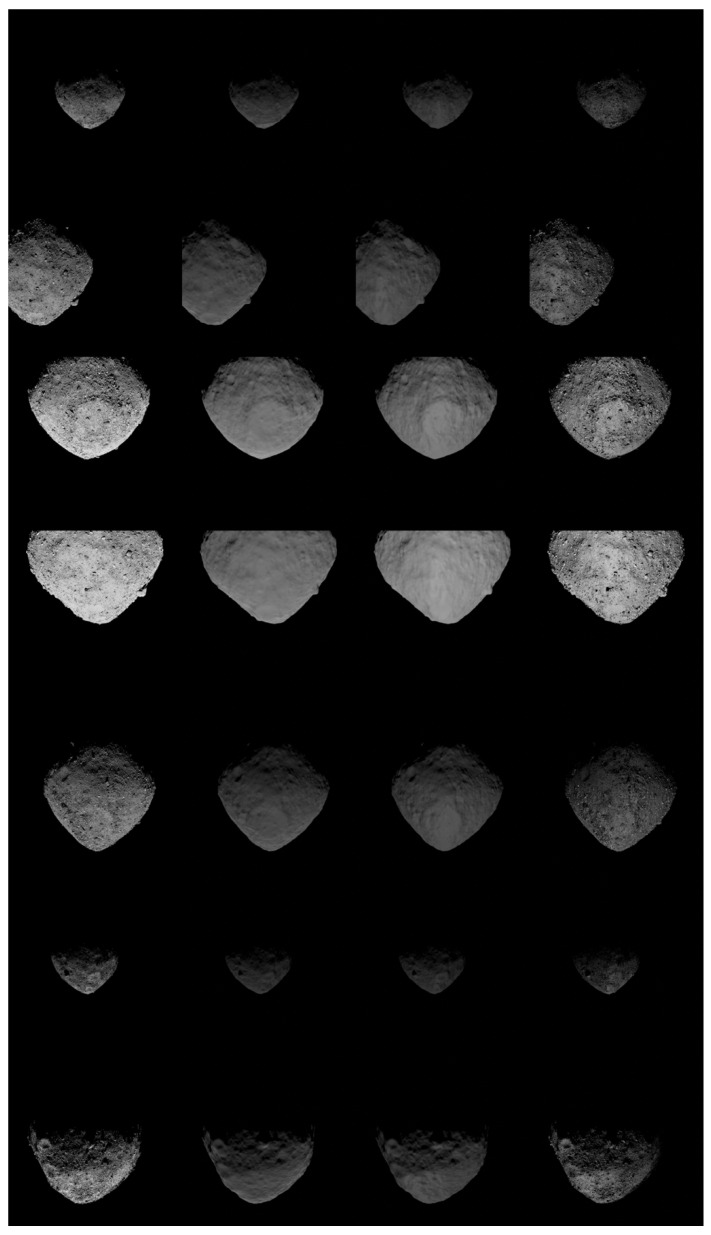
Mosaic view of Bennu. The first column represents real images, while, progressing from the second to the last column, synthetically generated images are depicted using CORTO with the OSL reflectance models, PBSDF, and PBSDF + Texture, respectively.

The validation results of the pipeline over these bodies are presented both in a quantitative way in Table 3 and Table 4 using the SSIM similarity metric, and in a visual way in Figure 16 and Figure 17. The link between the tables and the mosaic views is represented by the row, and column coordinates listed in the last column of each table. Moreover, Table 3 and Table 4 provide details about each sample, including the original name of the image, the key rendering properties used (the scattering function, from 0 associated with the PBSDF, to 6 following the order illustrated in Section 2.2, albedo, and Sun’s intensity), the noise combination (expressed as 4 components, respectively, for Gaussian mean and variance, blur, and Brigthness as in Table 1), and the associated SSIM with the most similar synthetic image.

From the results of the validation pipeline, from a qualitative inspection it is possible to determine that CORTO can represent the target bodies as realistic input for image processing and visual-based applications. Remarkably, the use of texture maps yields mixed results for the cases of Ceres and Bennu. In particular, considering all the reflectance strategies for Bennu, the one using “PBSDF + Texture” turns out to be the one returning the highest similarity scores, as can be seen by the higher values of SSIM in Table 4. On the other hand, the values of SSIM for the case of Ceres are lower than expected, even when using a texture map over the surface. This difference is ascribed to the quality of the available textures and the data they represent (e.g., mostly craters and albedo variations for Ceres and boulders and albedo variations for Bennu). This indicates that whenever this information is available at high resolution, it can significantly improve the similarity of synthetic images. Moreover, the effect is more relevant when representing boulder fields over plain and cratered regions. Unfortunately, this form of data is solely available at high resolution and with the correct illumination conditions only for a limited number of bodies. Nonetheless, when texture maps are not used, as in the case of Vesta and 67P, CORTO can represent the appearance of the bodies at a global level with a reasonable level of fidelity.

## 4. Case Studies

Due to its capabilities, CORTO has been concurrently developed by the authors and used within the DART group since the summer of 2020 in a variety of research activities, projects, and missions.

CORTO has been extensively used and co-developed for the design and validation of the image processing and visual-based GNC of the Milani CubeSat [46], a 6U CubeSat that will visit the Didymos binary system in 2027. Milani is an ESA mission that is part of the Hera mission [47]. The tool proved to be critical in the design of the data-driven algorithms within the image processing of Milani, in the testing of the object recognition algorithm, and in validating all the visual-based applications of the GNC subsystem of the CubeSat.

CORTO capabilities have also been used to test the limb-based navigation around the Moon for the LUMIO mission [48]. LUMIO is a 12U CubeSat that will orbit in a Halo orbit about the Earth-Moon L2 point and is an ASI/ESA mission. LUMIO will perform a scientific investigation about meteoroid impacts and act as a technology demonstrator for visual-based navigation techniques.

Additionally, up-to-date CORTO is currently used in different ASI and ESA projects, most notably the DeepNav [49] and StarNav projects. DeepNav is currently investigating the design and implementation of deep-learning techniques for visual-based navigation around small bodies using onboard processors. All the datasets used within the DeepNav project for the training and testing of the deep-learning methods have been generated with CORTO and using a HIL setup with the TinyV3RSE facility. Finally, StarNav is an ongoing project investigating star trackers’ image-processing capabilities for close proximity operations about asteroids and the Moon. The test images for the image-processing algorithms have been generated using CORTO.

## 5. Conclusions and Future Implementations

In conclusion, CORTO is a tool that provides a comprehensive and versatile platform for generating synthetic images of celestial bodies, facilitating the development and validation of image processing and navigation algorithms for space missions. Its capabilities span rendering, noise modeling, hardware-in-the-loop testing, and post-processing, enabling researchers and engineers to simulate realistic scenarios and assess algorithm performance. The validation pipeline, utilizing metrics like normalized cross-correlation and structural similarity via SSIM, ensures the tool’s accuracy and reliability compared to images from previously flown missions. CORTO has demonstrated its utility in various case studies and projects, including CubeSat design, lunar missions, and deep learning applications. CORTO represents a valuable asset in advancing space exploration and navigation capabilities through effective algorithm development and validation.

While the tool covers various aspects of celestial body simulation, future enhancements could extend its scope to incorporate additional phenomena and planetary environments. The tool is completely focused on visible images. However, it would be interesting to extend its applicability to cover thermal and infrared sensors. CORTO has been primarily developed and designed for minor bodies such as asteroids and comets. However, the tool’s usage has been recently expanded for Moon scenarios. Future implementations could cover other planets of interest, such as Mars and Venus, requiring high-fidelity modeling of atmospheric effects, which is currently not implemented. The same type of modeling would be of interest for a comet’s coma. Additionally, CORTO currently represent environments useful for close proximity operations about celestial bodies. However, future implementation could focus on terrain simulations for lander and rover Earth-based, visual-based applications. Also, it is highlighted that currently, the tool is not designed for real-time simulations. This is a functionality that could be investigated in future versions.

## Figures and Tables

**Table 1:** Considered noise values to be applied to the *M* template images.

Noise Type	Values
Gaussian mean	0.01,0.09,0.17,0.25
Gaussian variance	$10^{-5},10^{-4},10^{-3}$
Blur	0.6,0.8,1.0,1.2
Brightness	1.00,1.17,1.33,1.50

**Table 2:** Number of template images for each combination of target body and scattering function used to represent the surface.

	OSL	PBSDF	PBSDF + Texture
Ceres	-	-	693
Vesta	8316	-	-
67P	1512	-	-
Bennu	2646	686	819

**Table 3:** Properties of the synthetic images of comet 67P and asteroids Ceres and Vesta. The first four rows correspond to 67P, five to twelve represent Ceres, and thirteen to twenty refer to Vesta. The last column represents the position as (row, column) coordinates of the synthetic image in Figure 16.

Img Name	Rendering	Noise	SSIM	ID
N20160128T002344268ID20F71	4,0.15,40	$0.01,10^{-5},1.2,1.00$	0.7537	1,1
N20160130T173323717ID20F22	6,0.15,30	$0.01,10^{-5},1.2,1.00$	0.4421	1,3
W20150316T053347931ID20F13	3,0.5,40	$0.09,10^{-5},1.2,1.50$	0.9360	2,1
W20160617T102200832ID20F18	5,0.5,10	$0.09,10^{-5},1.2,1.33$	0.8920	2,3
FC21A0037273_15136172940F1E	0,−,4.25	$0.09,10^{-3},0.8,1.00$	0.4430	3,1
FC21A0037405_15157034032F3I	0,−,4.20	$0.09,10^{-3},0.6,1.50$	0.3979	3,3
FC21A0037589_15158013232F1I	0,−,3.25	$0.09,10^{-3},0.8,1.00$	0.3558	4,1
FC21A0037593_15158020232F1I	0,−,6.50	$0.09,10^{-3},0.8,1.00$	0.4973	4,3
FC21A0037978_15163064254F1G	0,−,6.25	$0.09,10^{-3},0.6,1.50$	0.2870	5,1
FC21A0038693_15172150728F6G	0,−,6.00	$0.09,10^{-3},0.6,1.50$	0.3557	5,3
FC21A0038787_15173122643F1G	0,−,1.50	$0.01,10^{-5},1.2,1.00$	0.3379	6,1
FC21A0039042_15176210244F1H	0,−,2.50	$0.09,10^{-3},0.8,1.17$	0.6744	6,3
FC21B0003258_11205095604F6C	6,0.5,3	$0.09,10^{-3},0.8,1.17$	0.7201	7,1
FC21B0003428_11205235222F5C	6,0.5,2	$0.09,10^{-3},0.8,1.17$	0.8499	7,3
FC21B0003757_11218102757F7D	3,0.5,2	$0.09,10^{-3},0.8,1.00$	0.7034	8,1
FC21B0003866_11218121551F4D	5,0.5,3	$0.09,10^{-3},0.6,1.50$	0.8337	8,3
FC21B0004630_11226232738F7D	6,0.5,2	$0.09,10^{-3},0.6,1.33$	0.6480	9,1
FC21B0005299_11230130409F6B	6,0.5,1	$0.09,10^{-3},0.8,1.17$	0.8114	9,3
FC21B0005871_11232204234F4B	2,0.5,2	$0.01,10^{-3},1.2,1.50$	0.6644	10,1
FC21B0006422_11238100914F1B	6,0.5,1	$0.01,10^{-4},1.0,1.33$	0.8142	10,3

**Table 4:** Properties of the synthetic images of Bennu. For every real image, presented in the first column, three rows provide information about the corresponding synthetic image properties, namely, OSL, PBSDF, and PBSDF + Texture. The last column represents the coordinate as (row, column) of the synthetic image in Figure 17.

Img Name	Rendering	Noise	SSIM	ID
20181211T181336S699_map_specradL2b	6,0.15,40	$0.01,10^{-4},1.0,1.33$	0.9200	1,2
	0,−,0.40	$0.01,10^{-4},1.0,1.33$	0.9187	1,3
	0,−,3.75	$0.01,10^{-4},0.8,1.00$	0.9458	1,4
20181212T043459S572_map_specradL2x	6,0.50,20	$0.01,10^{-4},1.0,1.33$	0.8054	2,2
	0,−,0.60	$0.01,10^{-4},1.0,1.33$	0.8046	2,3
	0,−,6.00	$0.01,10^{-4},0.8,1.00$	0.8958	2,4
20181212T064255S344_map_radL2pan	6,0.50,40	$0.01,10^{-4},1.0,1.33$	0.7090	3,2
	0,−,1.20	$0.01,10^{-4},1.0,1.33$	0.7078	3,3
	0,−,14.75	$0.01,10^{-4},0.8,1.00$	0.8549	3,4
20181212T085936S404_map_iofL2pan	6,0.50,40	$0.01,10^{-4},1.0,1.33$	0.7131	4,2
	0,−,2.00	$0.01,10^{-4},1.0,1.33$	0.7165	4,3
	0,−,23.5	$0.01,10^{-4},0.8,1.00$	0.8459	4,4
20181213T043620S487_map_radL2pan	6,0.20,40	$0.01,10^{-4},1.0,1.33$	0.7537	5,2
	0,−,0.60	$0.01,10^{-4},0.8,1.50$	0.7528	5,3
	0,−,5.00	$0.01,10^{-4},0.8,1.00$	0.8175	5,4
20181215T053926S725_map_iofL2b	3,0.15,35	$0.01,10^{-4},1.0,1.33$	0.9339	6,2
	0,−,0.30	$0.01,10^{-4},1.0,1.33$	0.9337	6,3
	0,−,3.00	$0.01,10^{-4},0.8,1.00$	0.9473	6,4
20181217T033612S897_map_iofL2pan	6,0.45,20	$0.01,10^{-4},1.0,1.33$	0.8127	7,2
	0,−,0.60	$0.01,10^{-4},1.0,1.33$	0.8107	7,3
	0,−,6.50	$0.01,10^{-4},0.8,1.00$	0.8845	7,4

## Data Availability

Data available on demand.

## Abbreviations

The following abbreviations are used in this manuscript:

CORTO	Celestial Object Rendering TOol
DART	Deep-Space Astrodynamic Research and Technology
GNC	Guidance Navigation and Control
HIL	Hardware-In-the-Loop
IP	Image Processing
MONET	Minor bOdy GeNErator Tool
NRMSE	Normalized Root Mean Square Error
OSL	Open Shading Language
PBSDF	Principled Bi-directional Scatter Distribution Function
SSIM	Structural Similarity Index Measure
TinyV3RSE	Tiny Versatile 3D Reality Simulation Environment

## Appendix A

The post-processing pipeline depicted in Figure 12 implements a series of steps that are part of a data augmentation strategy designed explicitly for celestial bodies and their interest labels. Traditional data augmentation strategies are limiting the capabilities to transform the image–label pairs in a useful way. In this Appendix, the transformations to change labels, such as the center of brightness (CoB), the correction vector between the estimated center of mass (CoF) and the CoB (δ), the range ρ, the phase angle (Ψ), and the Position of the camera ([X,Y,Z]), and the equatorial and elevation angles ($\left[\Phi_{1},\Phi_{2}\right]$) are presented. For example, performing the $S_{0}\to S_{1}$ transformation from $S_{0}$ to $S_{1}$ results in the following set of changes:

(A1) $${\mathrm{CoB}}^{S_{1}}={\mathrm{CoB}}^{S_{0}}-\left[\begin{array}{c} \Gamma_{1}\\ \Gamma_{2} \end{array}\right]+\left[\begin{array}{c} \alpha_{u}\\ \alpha_{v} \end{array}\right]$$

(A2) $${\mathrm{CoF}}^{S_{1}}={\mathrm{CoF}}^{S_{0}}-\left[\begin{array}{c} \Gamma_{1}\\ \Gamma_{2} \end{array}\right]+\left[\begin{array}{c} \alpha_{u}\\ \alpha_{v} \end{array}\right]$$

(A3) $$\delta^{S_{1}}=\delta^{S_{0}}$$

(A4) $$\rho^{S_{1}}=\rho^{S_{0}}$$

(A5) $${\left[X,Y,Z\right]}^{S_{1}}={\left[X,Y,Z\right]}^{S_{0}}$$

(A6) $${\left[\phi_{1},\phi_{2}\right]}^{S_{1}}={\left[\phi_{1},\phi_{2}\right]}^{S_{0}}$$

(A7) $$\Psi^{S_{1}}=\Psi^{S_{0}}$$

Performing the $S_{1}\to S_{2}$ transformation from $S_{1}$ to $S_{2}$ the labels are transformed as follows:

(A8) $${\mathrm{CoB}}^{S_{2}}={\mathrm{CoB}}^{S_{1}}\frac{128}{\gamma }$$

(A9) $${\mathrm{CoF}}^{S_{2}}={\mathrm{CoF}}^{S_{1}}\frac{128}{\gamma }$$

(A10) $$\delta^{S_{2}}=\delta^{S_{1}}\frac{128}{\gamma }$$

(A11) $$\rho^{S_{2}}=\rho^{S_{1}}\frac{128}{\gamma }$$

(A12) $${\left[X,Y,Z\right]}^{S_{2}}={\left[X,Y,Z\right]}^{S_{1}}\frac{128}{\gamma }$$

(A13) $${\left[\phi_{1},\phi_{2}\right]}^{S_{2}}={\left[\phi_{1},\phi_{2}\right]}^{S_{1}}$$

(A14) $$\Psi^{S_{2}}=\Psi^{S_{1}}$$

where γ, $\Gamma_{1}$, $\Gamma_{2}$, $\alpha_{u}$, $\alpha_{v}$, ${CoB}_{u}^{S_{0}}$, and ${CoB}_{v}^{S_{0}}$ are extracted during the execution of the post-processing pipeline and are values that can be easily stored. Their meaning is fully explained in Section 2.5.

The concatenation between the transformations $S_{0}\to S_{1}$ and $S_{1}\to S_{2}$ and viceversa is straightforward.

Finally, as image–label pairs are often used to estimate a position with respect to the target body, the necessary transformations to transform the labels illustrated above into positions are briefly described. When an image is used to extract the cartesian coordinates [X,Y,Z] in $S_{2}$ in any reference frame, these are transformed back to $S_{0}$ as:

(A15) $$p_{est}^{S_{0}}=\frac{\gamma }{128}{\left[\begin{array}{c} X\\ Y\\ Z \end{array}\right]}_{est}^{S_{2}}$$

On the other hand, the same transformation using spherical coordinates is:

(A16) $$p_{est}^{S_{0}}=\Omega \left({\left[\begin{array}{c} \phi_{1}\\ \phi_{2}\\ \rho \frac{\gamma }{128} \end{array}\right]}_{est}^{S_{2}}\right)$$

where Ω represents the trivial transformation function from spherical to cartesian coordinates. Finally, any IP method working in inference with δ and ρ labels requires a set of intermediate steps to generate a position estimate. The following optical observations are generated in inference in $S_{0}$:

(A17) $$o_{est}^{uv,S_{0}}=\left[\begin{array}{c} {CoF}_{est,u}^{S_{0}}\\ {CoF}_{est,v}^{S_{0}}\\ 1 \end{array}\right]=\left[\begin{array}{c} {CoB}_{u}^{S_{0}}+{\delta_{u}}^{S_{2}}\frac{\gamma }{128}\\ {CoB}_{v}^{S_{0}}+{\delta_{v}}^{S_{2}}\frac{\gamma }{128}\\ 1 \end{array}\right]$$

(A18) $$\rho_{est}^{S_{0}}=\rho^{S_{2}}\frac{\gamma }{128}$$

where $\delta_{u}^{S_{2}}$, $\delta_{v}^{S_{2}}$, and $\rho^{S_{2}}$ are the output of the IP method while all other variables are computed and stored during the processing of the image by the pipeline. The vector of optical observables $o_{est}^{uv,S_{0}}$ is then transformed from the image reference frame, which expresses pixel coordinates on the image to the IMP reference frame using the inverse of the camera calibration matrix $K^{-1}$ defined with the notation in [50]:

(A19) $$o_{est}^{ImP}=K^{-1}o_{est}^{uv,S_{0}}$$

The $o_{est}^{ImP}$ vector is then transformed into a LOS vector in the camera reference frame. Using the range $\rho^{S_{2}}$, this LOS can be transformed into a position estimate in the camera reference frame $p_{est}^{\mathrm{CAM}}$. Knowing the appropriate transformations, this position can be transformed in the reference frame of interest.
